# Intermittent Hypoxia and Hypercapnia Alter Diurnal Rhythms of Luminal Gut Microbiome and Metabolome

**DOI:** 10.1128/mSystems.00116-21

**Published:** 2021-06-29

**Authors:** Celeste Allaband, Amulya Lingaraju, Cameron Martino, Baylee Russell, Anupriya Tripathi, Orit Poulsen, Ana Carolina Dantas Machado, Dan Zhou, Jin Xue, Emmanuel Elijah, Atul Malhotra, Pieter C. Dorrestein, Rob Knight, Gabriel G. Haddad, Amir Zarrinpar

**Affiliations:** a Division of Gastroenterology, University of California, San Diego, La Jolla, California, USA; b Biomedical Sciences Graduate Program, University of California, San Diego, La Jolla, California, USA; c Department of Pediatrics, University of California, San Diego, La Jolla, California, USA; d Bioinformatics and Systems Biology Program, University of California, San Diego, La Jolla, California, USA; e Collaborative Mass Spectrometry Innovation Center, Skaggs School of Pharmacy, University of California, San Diego, La Jolla, California, USA; f Department of Neuroscience, University of California, San Diego, La Jolla, California, USA; g Center for Microbiome Innovation, University of California, San Diego, La Jolla, California, USA; h Department of Computer Science and Engineering, University of California, San Diego, La Jolla, California, USA; i Institute of Diabetes and Metabolic Health, University of California, San Diego, La Jolla, California, USA; j Center for Circadian Biology, University of California, San Diego, La Jolla, California, USA; k VA Health Sciences San Diego, La Jolla, California, USA; Columbia University Irving Medical Center

**Keywords:** circadian rhythm, metabolome, microbiome, animal models of human disease, atherosclerosis, computational biology

## Abstract

Obstructive sleep apnea (OSA), characterized by intermittent hypoxia and hypercapnia (IHC), affects the composition of the gut microbiome and metabolome. The gut microbiome has diurnal oscillations that play a crucial role in regulating circadian and overall metabolic homeostasis. Thus, we hypothesized that IHC adversely alters the gut luminal dynamics of key microbial families and metabolites. The objective of this study was to determine the diurnal dynamics of the fecal microbiome and metabolome of *Apoe^−/−^* mice after a week of IHC exposure. Individually housed, 10-week-old *Apoe^−/−^* mice on an atherogenic diet were split into two groups. One group was exposed to daily IHC conditions for 10 h (Zeitgeber time 2 [ZT2] to ZT12), while the other was maintained in room air. Six days after the initiation of the IHC conditions, fecal samples were collected every 4 h for 24 h (6 time points). We performed 16S rRNA gene amplicon sequencing and untargeted liquid chromatography-mass spectrometry (LC-MS) to assess changes in the microbiome and metabolome. IHC induced global changes in the cyclical dynamics of the gut microbiome and metabolome. *Ruminococcaceae*, *Lachnospiraceae*, S24-7, and *Verrucomicrobiaceae* had the greatest shifts in their diurnal oscillations. In the metabolome, bile acids, glycerolipids (phosphocholines and phosphoethanolamines), and acylcarnitines were greatly affected. Multi-omic analysis of these results demonstrated that *Ruminococcaceae* and tauro-β-muricholic acid (TβMCA) cooccur and are associated with IHC conditions and that *Coriobacteriaceae* and chenodeoxycholic acid (CDCA) cooccur and are associated with control conditions. IHC significantly change the diurnal dynamics of the fecal microbiome and metabolome, increasing members and metabolites that are proinflammatory and proatherogenic while decreasing protective ones.

**IMPORTANCE** People with obstructive sleep apnea are at a higher risk of high blood pressure, type 2 diabetes, cardiac arrhythmias, stroke, and sudden cardiac death. We wanted to understand whether the gut microbiome changes induced by obstructive sleep apnea could potentially explain some of these medical problems. By collecting stool from a mouse model of this disease at multiple time points during the day, we studied how obstructive sleep apnea changed the day-night patterns of microbes and metabolites of the gut. Since the oscillations of the gut microbiome play a crucial role in regulating metabolism, changes in these oscillations can explain why these patients can develop so many metabolic problems. We found changes in microbial families and metabolites that regulate many metabolic pathways contributing to the increased risk for heart disease seen in patients with obstructive sleep apnea.

## INTRODUCTION

Obstructive sleep apnea (OSA) is a major risk factor for cardiovascular disease (CVD), including metabolic syndrome, insulin resistance, cardiac arrhythmias, and atherosclerosis (1). The mechanism(s) of how OSA, or its characteristic components, intermittent hypoxia and hypercapnia (IHC), increases CVD risks is poorly understood, but disruption of circadian rhythms has long been suspected (2–4). The dyssynchrony between central and peripheral clock machineries could explain why IHC is able to disrupt so many different physiological systems simultaneously (5–7). OSA can affect the central circadian clock through sleep fragmentation and increased sympathetic tone. However, how OSA affects the agents that entrain peripheral circadian clocks is poorly understood.

Hepatic and intestinal circadian rhythms are entrained by feeding/fasting cycles and the gut microbiome (7, 8). The gut microbiome is necessary for the maintenance of ileal and hepatic circadian clocks and their synchrony with central circadian rhythms (9, 10). Moreover, the gut microbiome itself has cyclical fluctuations that are necessary for metabolic homeostasis (9–15). Microbially produced compounds, such as short-chain fatty acids (SCFAs) (16) as well as deconjugated and secondary bile acids (17), link the luminal environment with host hepatic and ileal circadian rhythms (8). Given that peripheral circadian rhythms regulate circulating lipids (18), hematopoietic stem cells (19), vascular smooth muscle function, sympathetic tone, and blood pressure (20, 21), OSA-induced changes to the gut microbiome could aggravate multiple physiological systems that promote atherosclerosis through their disruption. Moreover, disrupted circadian luminal dynamics can affect gut microniches and promote the growth of bacteria that are proinflammatory (e.g., *Ruminococcaceae* [22]) and hinder those that may be protective against CVD (e.g., *Akkermansia* [23]). Disruption of microbiome rhythms can also increase systemic inflammation through disruption of the gut barrier function (24). Thus, the effect of IHC on luminal diurnal dynamics can improve our understanding of how OSA increases CVD risks.

Prolonged IHC exposure in atherosclerotic mouse models results in the faster development and increased extent of atherosclerotic lesions, making them the preferred animal models of OSA. Interestingly, IHC exposure also alters the composition of the gut microbiome and fecal metabolome in both apolipoprotein E knockout (*Apoe^−/−^*) and low-density lipoprotein (LDL) receptor knockout (*Ldlr^−/−^*) mice on atherogenic diets (25, 26), findings that go beyond what is observed in these mice alone (27, 28). Moreover, the fecal metabolomic changes observed in these mice included metabolites known to affect atherosclerosis, including trimethylamine (TMA), deconjugated and secondary bile acids, fatty acids, and phytoestrogens (25, 26, 29). However, some of these luminal metabolites have diurnal fluctuations and are differentially absorbed based on the enterocyte circadian clock (30, 31). Characterizing the time resolution of these changes would further our understanding of how luminal content could contribute to dysmetabolism and atherosclerosis.

To focus solely on the effects of IHC conditions on the diurnal dynamics of the gut microbiome and metabolome, we maintained consistent genotype (*Apoe^−/−^*) and dietary conditions (atherogenic diet) between groups. We wanted to evaluate whether atherogenic gut luminal changes persist through a 24-h period or only during certain windows of time. Overall, this study tests the hypothesis that IHC disrupts the diurnal rhythms of the gut microbiome and metabolome, which may promote a proinflammatory luminal environment.

## RESULTS

### IHC change the composition of the gut microbiome.

**FIG 1:**
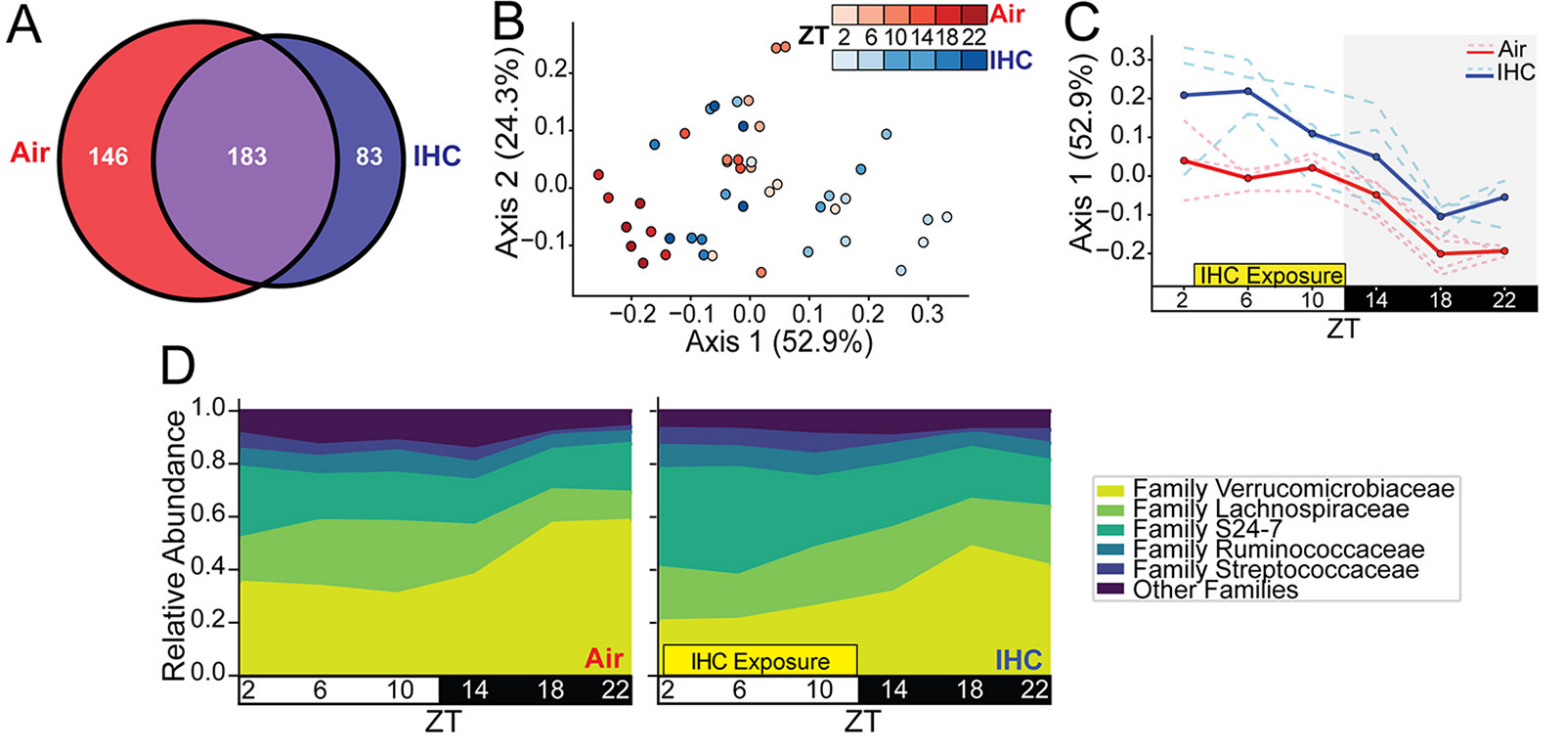
IHC affects the cyclical dynamics of the gut microbiome. (A) Venn diagram of unique nonzero detected sOTUs in each cohort overall. Purple indicates sOTUs in common. (B) Weighted UniFrac (beta diversity) PCoA of samples. Shading represents different time points as indicated. (C) Weighted UniFrac PCoA of only axis 1 over time. The solid lines indicate the average for the group, and the dotted lines indicate individual mice. (D) Proportional-abundance representation of the top 5 microbial families. Control samples exposed only to normal air conditions are in red (*n* = 4; 5 to 6 time points per mouse). Experimental samples exposed to IHC conditions are in blue (*n* = 4; 5 to 6 time points per mouse).

To determine the early effects of OSA on the gut microbiome, we used the atherosclerosis-prone *Apoe^−/−^* murine model and exposed half of the cohort to IHC conditions, while control mice were exposed to room air. Since mice were individually housed for the experiment, cage effects were not a confounding variable. Our analysis revealed a large overlap in the sub-operational taxonomic units (sOTUs) (Fig. 1A), with 183 sOTUs in common between the two groups. These shared sOTUs comprised 56% of the total air sOTUs and 69% of the total IHC sOTUs. The air control group tended to have more sOTUs than the IHC group (*P* = 0.053 by a Mann-Whitney U test) (see Fig. S1A in the supplemental material). In addition, mice under the air conditions had overall higher Faith’s phylogenetic α-diversity than mice under the IHC conditions (*P* = 0.040 by a Kruskal-Wallis test) (Fig. S1B). This finding is especially true for Zeitgeber time 18 [ZT18], where α-diversity values diverged the most between the two groups (*P* = 0.015 by a Mann-Whitney U test). Gut microbiome biodiversity, such as that measured by Faith’s phylogenetic diversity, has previously been used as a surrogate measure of microbial community health (32).

10.1128/mSystems.00116-21.1 FIG S1 Overview of the 16S fecal microbiome of ApoE^−/−^ mice on an atherogenic diet with IHC treatment. (A) Alpha rarefaction curve. The dotted line indicates 12,000 (rarefaction depth). Shaded regions indicate 95% confidence intervals. A Mann-Whitney U test was used to determine statistical significance (statistic = 190.5; *P* value = 0.053). (B) Faith’s phylogenetic alpha diversity over time for each sample, colored by group. Shaded regions indicate standard errors of the means. A Mann-Whitney-Wilcoxon test was used to determine statistical significance. (C) Weighted UniFrac PCoA, with samples highlighted by time point. (D) Pie graphs of cycling and noncycling microbes classified to at least the family level under both conditions. (E) Pie graphs of cycling and noncycling reads attributed to a family-level identifier under both conditions. Download FIG S1, TIF file, 1.4 MB. Copyright © 2021 Allaband et al. 2021 Allaband et al. https://creativecommons.org/licenses/by/4.0/ This content is distributed under the terms of the Creative Commons Attribution 4.0 International license.

Weighted UniFrac distances, a measure of β-diversity, were significantly different between the two groups across all time points (Fig. 1B; Fig. S1C) (pseudo-*F*, 6.776; *P* = 0.002 by permutational multivariate analysis of variance [PERMANOVA]) (33). Analysis of these β-diversity distances demonstrates that the microbiome composition of the IHC in the dark period became more similar to that of the air controls during the light period (Fig. 1B and C). Compositional analysis showed that *Verrucomicrobiaceae*, *Lachnospiraceae*, and S24-7 were the top three most prevalent taxa of the microbiome under both conditions across all time points (Fig. 1D). Family S24-7 (“*Homeothermaceae*” is one of the currently proposed names) is a relatively new family in the phylum *Bacteroidetes*, thought to be involved in carbohydrate metabolism, among other functions (34). To determine whether IHC disrupted the diurnal dynamics of the luminal environment, we determined the proportion of bacterial families that had diurnal oscillation in their abundance (*P* < 0.05) (35). Compared to control mice, the mice under the IHC conditions had about two-thirds as many bacterial families (air, 13%; IHC, 8%) that had diurnal oscillations (Fig. S1D), and these families accounted for fewer reads (Fig. S1E). See Table S3 in the supplemental material for a full breakdown of changes in oscillation at the sOTU level. Thus, IHC caused a significant change in luminal dynamics over the course of 24 h. This shift in luminal dynamics is characterized by a decrease in overall cycling, wherein the dark-period microbial composition in IHC is more similar to that of the light period in control mice. We did not see significant changes in rhythmicity (36).

10.1128/mSystems.00116-21.8 TABLE S3 Breakdown of cyclical oscillators (MetaCycle, JTK_CYCLE [*P* < 0.05]). Download Table S3, XLSX file, 0.01 MB. Copyright © 2021 Allaband et al. 2021 Allaband et al. https://creativecommons.org/licenses/by/4.0/ This content is distributed under the terms of the Creative Commons Attribution 4.0 International license.

### IHC exposure results in diurnal disruption of the gut microbiome.

To examine the effects of IHC on the diurnal dynamics of the microbiome in more detail, we examined individual taxa over time. IHC induced dynamic changes in the composition of the microbiome that were easily detectable at the phylum level (Fig. S2A to C). There was no detectable cyclical fluctuation in the *Bacteroidetes* phylum (Fig. S2A). Under the control conditions, *Firmicutes* had cyclical fluctuations with a peak in the late light period (Fig. S2B), and *Verrucomicrobia* had cyclical fluctuations with a peak in the late dark period (Fig. S2C). The phylum *Verrucomicrobia* contains only the sOTU for Akkermansia muciniphila. However, under IHC conditions, *Firmicutes* lost, and *Bacteroidetes* gained, cyclical oscillation (Fig. S2A and B). Cyclical oscillation was maintained in *Verrucomicrobia* under the IHC conditions (Fig. S2C). However, the relative abundances of this phylum were significantly higher in the air controls during both light and dark periods (Fig. S2C).

10.1128/mSystems.00116-21.2 FIG S2 IHC affects the cyclical dynamics of selected phyla and families in an OSA mouse model. 16S relative abundances of the phylum *Bacteroidetes* (A), the phylum *Firmicutes* (B), the phylum *Verrucomicrobia* (C), the family *Ruminococcaceae* (D), the family *Lachnospiraceae* (E), the family S24-7 (“*Homeothermaceae*”) (F), and the family *Coriobacteriaceae* (G) are shown. Solid lines represent the means, and error bars indicate standard errors of the means. Individual mice are indicated by dashed-line tracings. Shading indicates when room lights are off (i.e., active/feeding time for the mice). Yellow squares indicate the 10 h of IHC exposure under the IHC conditions. @, cyclical oscillation present (*P* < 0.05 as measured by MetaCycle [35] with the JTK method); *, *P* < 0.05 by a Mann-Whitney-Wilcoxon test. Control samples with exposure to only normal room air conditions are in red (*n* = 4; 5 to 6 time points per mouse). Experimental samples exposed to IHC conditions for 10 h per day are in blue (*n* = 4; 5 to 6 time points per mouse). Download FIG S2, TIF file, 2.4 MB. Copyright © 2021 Allaband et al. 2021 Allaband et al. https://creativecommons.org/licenses/by/4.0/ This content is distributed under the terms of the Creative Commons Attribution 4.0 International license.

Next, we analyzed the cyclical dynamics of *Ruminococcaceae* and *Lachnospiraceae*, two bacterial families in the *Firmicutes* phylum that have been associated with atherosclerosis formation (22). Both *Ruminococcaceae* (Fig. S2D) and *Lachnospiraceae* (Fig. S2E) have cyclical oscillations in the control mice that are perturbed under the IHC conditions. S24-7, a bacterial family in the *Bacteroidetes* phylum, was the only one that had more robust cycling under the IHC conditions than it did under the control conditions (Fig. S2F). In particular, S24-7 abundances increased significantly during the time of IHC exposure, where they were 2- to 3-fold higher than what was measured under the control conditions. In contrast, peak differences in the relative abundances of *Lachnospiraceae* occurred during the dark period, where abundances were approximately 2-fold higher under the IHC conditions. Overall, IHC perturbed the cyclical dynamics of the bacterial families that can affect the atherosclerosis phenotype.

### IHC change the composition of the fecal metabolome.

**FIG 2:**
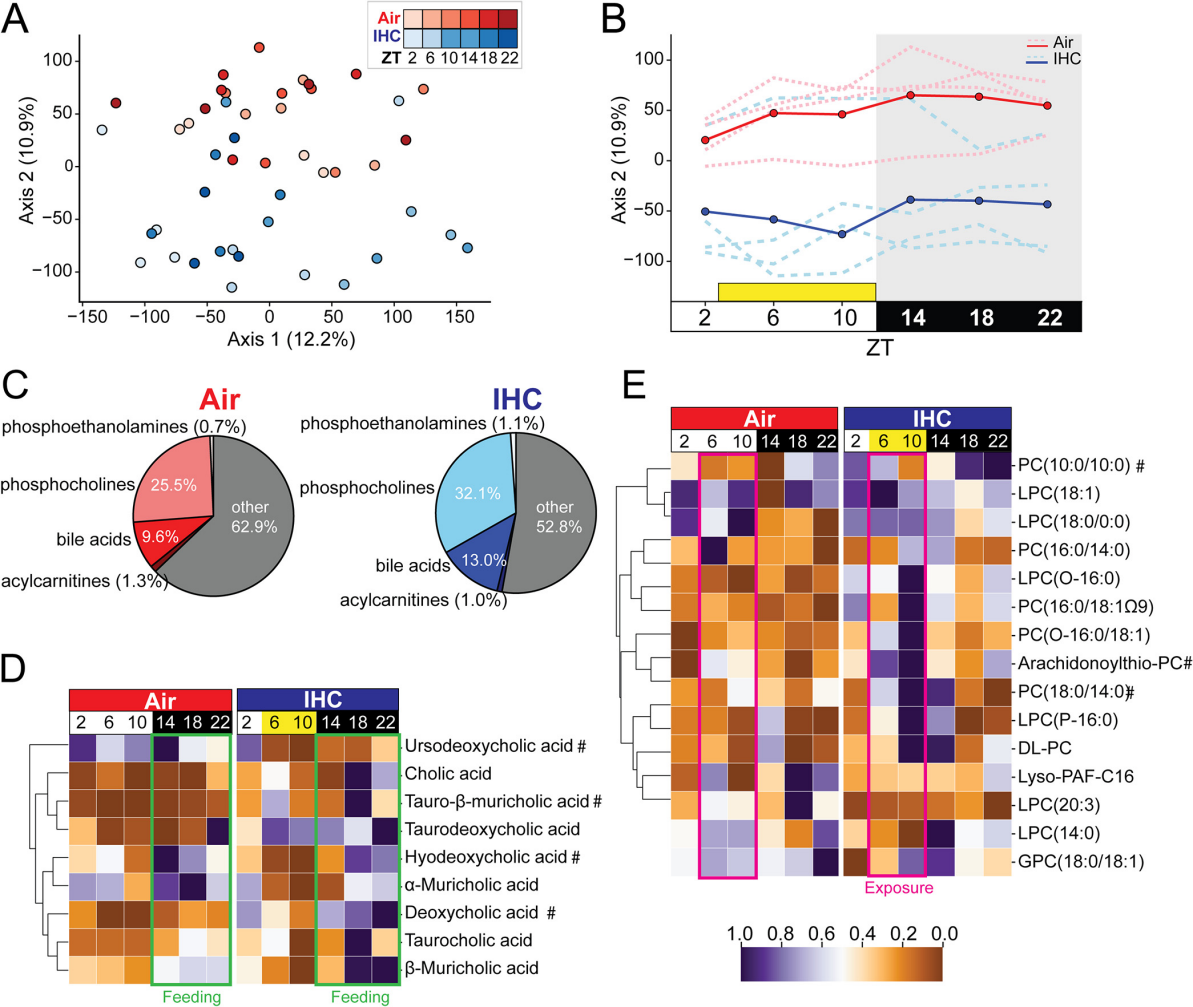
IHC affects the cyclical dynamics of the fecal metabolome. See Table S1 in the supplemental material for a list of the full annotations and abbreviations of the metabolites displayed. (A) Canberra PCoA of metabolomics samples. Shading represents different time points. Significance was determined by PERMANOVA. (B) Canberra PCoA of axis 2 over time. Solid lines indicate means for the group, and dotted lines indicate individual mice. The yellow box indicates the time under IHC exposure for the treatment group. (C) Pie charts of key groups of metabolites, separated by condition. (D) Heat map of level 1 bile acids, organized using hierarchical clustering based on controls. Yellow indicates the time under IHC exposure for the treatment group. For other level 3 bile acids, see Fig. S3C. (E) Heat map of selected phosphocholines, organized using hierarchical clustering based on controls. The value of each square of the heat map represents the mean relative abundance value for all mice under that condition for that time point. The heat maps are also row normalized across both conditions and placed on a standard scale, referenced in the center, to allow easier comparison. # indicates a metabolite that is also shown in Fig. 4 and Fig. S4. Air is in red (*n* = 4; 5 to 6 time points per mouse); IHC is in blue (*n* = 4; 5 to 6 time points per mouse).

An overview of the fecal metabolome, analyzed using untargeted liquid chromatography-tandem mass spectrometry (LC-MS/MS), shows significant separation between groups (pseudo-*F*, 5.410; *P* < 0.001 by PERMANOVA) (33) (Fig. 2A; Fig. S3A). Axis 2 (10.9%), which represents an amount of variability in the data nearly similar to that of axis 1 (12.2%), shows a clearer separation between groups over time (Fig. 2B). Overall, the IHC conditions led to an increase in the relative amounts of fecal bile acids (∼35%) as well as glycerolipids such as phosphoethanolamines (∼57%) and phosphocholines (∼26%) (Fig. 2C). The cyclical dynamics of these individual metabolites, which have been implicated in atherosclerosis, were significantly affected by IHC exposure (Fig. 2D and E; Fig. S3B) A list of all annotated metabolites can be found in Table S2 in the supplemental material.

10.1128/mSystems.00116-21.3 FIG S3 Additional untargeted LC-MS/MS metabolomics of fecal samples from ApoE^−/−^ mice on an atherogenic diet after 1 week of IHC treatment. (A) Heat map of additional selected metabolites. (B) Heat map of additional selected bile acids, including previously unrecognized bile acids. The value of each square of the heat map represents the average relative-abundance value (total sum normalized) for all mice under that condition for that time point. The heat maps are also row normalized (the row includes both conditions) and placed on a standard scale referenced on the left (0 [brown], lowest value; 1 [purple], highest value). The MS/MS spectral annotations were determined by using MS/MS-based spectral library matches for GNPS level 2 or 3 identification for all molecules displayed here. (C) Canberra PCoA with samples highlighted by time point. # indicates a metabolite that is shown in Fig. 2 and Fig. S4 in the supplemental material. Control samples exposed only to normal air conditions are in red (*n* = 4; 5 to 6 time points per mouse). Experimental samples exposed to IHC conditions are in blue (*n* = 4; 5 to 6 time points per mouse). Download FIG S3, TIF file, 2.2 MB. Copyright © 2021 Allaband et al. 2021 Allaband et al. https://creativecommons.org/licenses/by/4.0/ This content is distributed under the terms of the Creative Commons Attribution 4.0 International license.

10.1128/mSystems.00116-21.7 TABLE S2 All unique annotated metabolites. Download Table S2, TXT file, 0.01 MB. Copyright © 2021 Allaband et al. 2021 Allaband et al. https://creativecommons.org/licenses/by/4.0/ This content is distributed under the terms of the Creative Commons Attribution 4.0 International license.

10.1128/mSystems.00116-21.4 FIG S4 IHC affects the cyclical dynamics of bile acid fecal metabolites in an OSA mouse model. Untargeted LC-MS/MS was performed. Selected metabolites and their cyclical dynamics over time (longitudinal plot) (top) and their relative abundances grouped by cycle phase (box plots) (bottom) are shown. (A) Deoxycholic acid (DCA), level 1 identification. (B) Tauro-β-muricholic acid (TβMCA), level 1 identification. (C) Hyodeoxycholic acid (HDCA), level 1 identification. (D) Ursodeoxycholic acid (UDCA). (E) Allochenodeoxycholic acid (CDCA), level 3 annotation. Solid lines represent the means, and error bars indicate the standard errors of the means. Individual mice are indicated by dashed lines. Shading indicates when room lights are off (i.e., active/feeding time for the mice). Yellow squares indicate the 10 h of the day where mice under the IHC conditions would be exposed to experimental conditions (ZT2 to -12). @ indicates cyclical oscillations as determined by MetaCycle (35) (JTK) (*P* < 0.05). *, *P* < 0.05; **, *P* < 0.01; ***, *P* < 0.001; ****, *P* < 0.0001 (by a Mann-Whitney-Wilcoxon test). Control samples exposed only to normal air conditions are in red (*n* = 4; 5 to 6 time points per mouse). Experimental samples exposed to IHC conditions are in blue (*n* = 4; 5 to 6 time points per mouse). Download FIG S4, TIF file, 2.0 MB. Copyright © 2021 Allaband et al. 2021 Allaband et al. https://creativecommons.org/licenses/by/4.0/ This content is distributed under the terms of the Creative Commons Attribution 4.0 International license.

10.1128/mSystems.00116-21.6 TABLE S1 Metabolomic results and abbreviations. The MS/MS spectral annotations were determined by using MS/MS-based spectral library matches for GNPS level 3 identification for all molecules, except for some of the bile acids, as noted. Standards were run using the same method as the one used for samples for level 1 identification. Download Table S1, XLSX file, 0.01 MB. Copyright © 2021 Allaband et al. 2021 Allaband et al. https://creativecommons.org/licenses/by/4.0/ This content is distributed under the terms of the Creative Commons Attribution 4.0 International license.

Subclass analysis of the fecal metabolites demonstrated that time was an important factor in metabolomic differences. For example, the greatest differences in bile acids between IHC-conditioned mice and air controls occurred in the dark period (Fig. 2D), except for ursodeoxycholic acid (UDCA), which demonstrated the greatest changes during the light period. Phosphocholines, phosphoethanolamines, and acylcarnitines, metabolites that play an important role in atherosclerosis, are also altered under the IHC conditions (37–42). IHC resulted in comparatively high levels of phosphocholines (Fig. 2C and E) and phosphoethanolamines (Fig. 2C; Fig. S3B) during IHC exposure (i.e., light period) (Fig. 2E; Fig. S3B). In addition, IHC mice also had higher levels of heme breakdown derivatives, stercobilin and urobilin, during the dark period (Fig. S3B), which may be an indication of altered liver metabolism (43). Overall, fecal IHC induced global changes in fecal metabolomics, particularly in secondary metabolites that are known to contribute to inflammation and atherosclerosis.

### Diurnal dynamics of the fecal metabolome are altered under IHC conditions.

We performed a more detailed analysis of the effects of IHC on the diurnal dynamics of the luminal metabolites that are presumed to either exacerbate or protect against atherosclerosis, affect circadian rhythms, or influence metabolic homeostasis. Bile acids are some of the key metabolites that can influence peripheral circadian rhythms, host metabolism, and atherosclerosis (44). Deoxycholic acid (DCA), a proinflammatory secondary bile acid (45), had diurnal oscillations under the IHC conditions but not under the control conditions (air, *P* = 1.000; IHC, *P* = 0.002) (Fig. S4A). The relative abundance of DCA was higher under IHC conditions than under control conditions at all time points but especially during the dark period (*P* = 0.001) (Fig. S4A). Tauro-β-muricholic acid (TβMCA), known to contribute to the development of atherosclerosis through farnesoid X receptor (FXR) antagonism (46–48), had overall higher relative abundances in mice under IHC conditions (Fig. S4B). Significantly high levels of TβMCA were observed in IHC-conditioned mice during the light period (*P* = 0.019), with dark-period differences only approaching statistical significance (*P* = 0.063). TβMCA appears to have 12-h oscillations rather than 24-h oscillations. While proatherosclerotic TβMCA did not have a cyclical oscillation under air or IHC conditions (air, *P* = 0.270; IHC, *P* = 1.000) (Fig. S4B), antiatherosclerotic hyodeoxycholic acid (HDCA) (49, 50) displayed diurnal oscillations under IHC but not under control conditions (air, *P* = 1.000; IHC, *P* = 0.002) (Fig. S4C). Levels of anti-inflammatory UDCA (49, 51, 52) trended toward being lower in mice under IHC conditions; however, this did not approach significance (Fig. S4D). Overall, bile acid differences between the IHC and air conditions were far more pronounced during the dark period (Fig. 2D). Furthermore, with increased levels of proinflammatory and proatherosclerotic bile acids and a reduction in anti-inflammatory bile acids, IHC conditions appear to shift the metabolome in a detrimental direction for the host.

IHC had a significant effect on the cyclical dynamics of other metabolites that are important for atherosclerosis, such as acylcarnitines and glycerolipids, including phosphoethanolamines and phosphocholines (Fig. S5). High serum levels of long-chain acylcarnitines such as palmitoylcarnitine (Fig. S5A) and oleoyl l-carnitine (Fig. S5B) can promote inflammation and atherosclerosis (53, 54). However, fecal palmitoylcarnitine levels are significantly lower in IHC-conditioned mice during both periods of the circadian cycle (light period, *P* = 0.013; dark period, *P* = 0.017) (Fig. S5A). Overall levels of oleoyl l-carnitine, another acylcarnitine, are also reduced in IHC-conditioned mice, particularly during the light period of the circadian rhythm (light period, *P* = 0.053; dark period, *P* = 0.761) (Fig. S5B). Phosphoethanolamines and phosphocholines are the main proinflammatory glycerolipid derivatives that are absorbed into the bloodstream (55). Fecal levels of phosphoethanolamines were generally increased in IHC-conditioned mice, especially during the light period (Fig. 2). Levels of one such phosphoethanolamine, LysoPE[18:1(9Z)/0:0] [1-(9Z-octadecenoyl)-*sn*-glycero-3-phosphoethanolamine], had robust diurnal oscillations (*P* < 0.001) under IHC conditions, with significantly higher levels during the light period (*P* < 0.001) (Fig. S5C). In addition, overall levels of phosphocholines were also increased in IHC-conditioned mice, especially during the light period (Fig. 2). The levels of one phosphocholine in our study, PC(18:0/14:0) (1-stearoyl-2-myristoyl-*sn*-glycero-3-phosphocholine), showed robust cycling under the IHC conditions but not under the air conditions (air, *P* = 1.000; IHC, *P* = 0.002) and were especially elevated during the light period (*P* = 0.013) (Fig. S5D). Similar trends are seen in other phosphocholines (Fig. S5E and F). Taken together, our results demonstrate that IHC induces a rapid shift in the gut luminal metabolite profile.

10.1128/mSystems.00116-21.5 FIG S5 IHC affects the cyclical dynamics of selected fecal metabolites in an OSA mouse model. Untargeted LC-MS/MS was performed. Selected metabolites and their cyclical dynamics over time (longitudinal plot) (top) and their relative abundances grouped by cycle phase (box plots) (bottom) are shown. (A) Palmitoylcarnitine, level 3 annotation. (B) 1-(9Z-Octadecenoyl)-*sn*-glycero-3-phosphoethanolamine {LysoPE[18:1(9Z)/0:0]}, level 3 annotation. (C) 1-Stearoyl-2-myristoyl-*sn*-glycero-3-phosphocholine [PC(18:0/14:0)], level 3 annotation. (D) Oleoyl l-carnitine, level 3 annotation. (E) 1-Hexadecyl-2-arachidonoylthio-2-deoxy-*sn*-glycero-3-phosphocholine (arachidonoylthio-PC), level 3 identification. (F) Didecanoyl-glycerophosphocholine [PC(10:0/10:0)], level 3 annotation. Solid lines represent the means, and error bars indicate the standard errors of the means. Individual mice are indicated by dashed lines. Shading indicates when room lights are off (i.e., active/feeding time for the mice). Yellow squares indicate the 10 h of the day where mice under the IHC conditions would be exposed to experimental conditions (ZT2 to -12). @ indicates cyclical oscillations as determined by MetaCycle (35) (JTK) (*P* < 0.05). *, *P* < 0.05; **, *P* < 0.01; ***, *P* < 0.001; ****, *P* < 0.0001 (by a Mann-Whitney-Wilcoxon test). Control samples exposed only to normal air conditions are in red (*n* = 4; 5 to 6 time points per mouse). Experimental samples exposed to IHC conditions are in blue (*n* = 4; 5 to 6 time points per mouse). Download FIG S5, TIF file, 2.5 MB. Copyright © 2021 Allaband et al. 2021 Allaband et al. https://creativecommons.org/licenses/by/4.0/ This content is distributed under the terms of the Creative Commons Attribution 4.0 International license.

### Trans-omic analysis of the microbiome and metabolome reveals a key relationship between *Ruminococcaceae* and TβMCA.

Next, we assessed whether there are specific relationships between microbial families and metabolites that are individually implicated in worsening CVD. One particular challenge in performing multi-omic analysis with microbiome data is that 16S amplicon sequencing yields sum constraint-normalized data (i.e., relative abundances). Thus, this increases the probability of type I errors in the analysis and makes measurements of false discovery rates (FDRs) difficult (56). Relative abundance values can fluctuate significantly from study to study due to artifactual differences in the total number of microbial reads (i.e., total feature load). For example, when the relative abundance of a specific bacterial family is increased, we cannot determine if this is due to an increase in the number of bacteria within that family or a decrease in the number of other bacteria in other families. By using log ratios for these analyses, we remove the biases created by the total feature load and can calculate false discovery rates using previously established methods (56, 57). Thus, these log-ratio-based methods are more likely to result in repeatable trends in independently performed studies.

We used a machine learning neural network to predict the probability of microbe-metabolite interactions (mmvec [58]) as well as a multinomial logarithmic regression differential ranking analysis (songbird [56]). This analysis created ranked log-based conditional probabilities of microbe and metabolite cooccurrences and identified relationships between the microbiome and metabolome data, which were then validated using the individual log ratios. This analysis revealed not only which microbes and metabolites cooccur but also whether they are correlated with differences between the IHC and control conditions.

**FIG 3:**
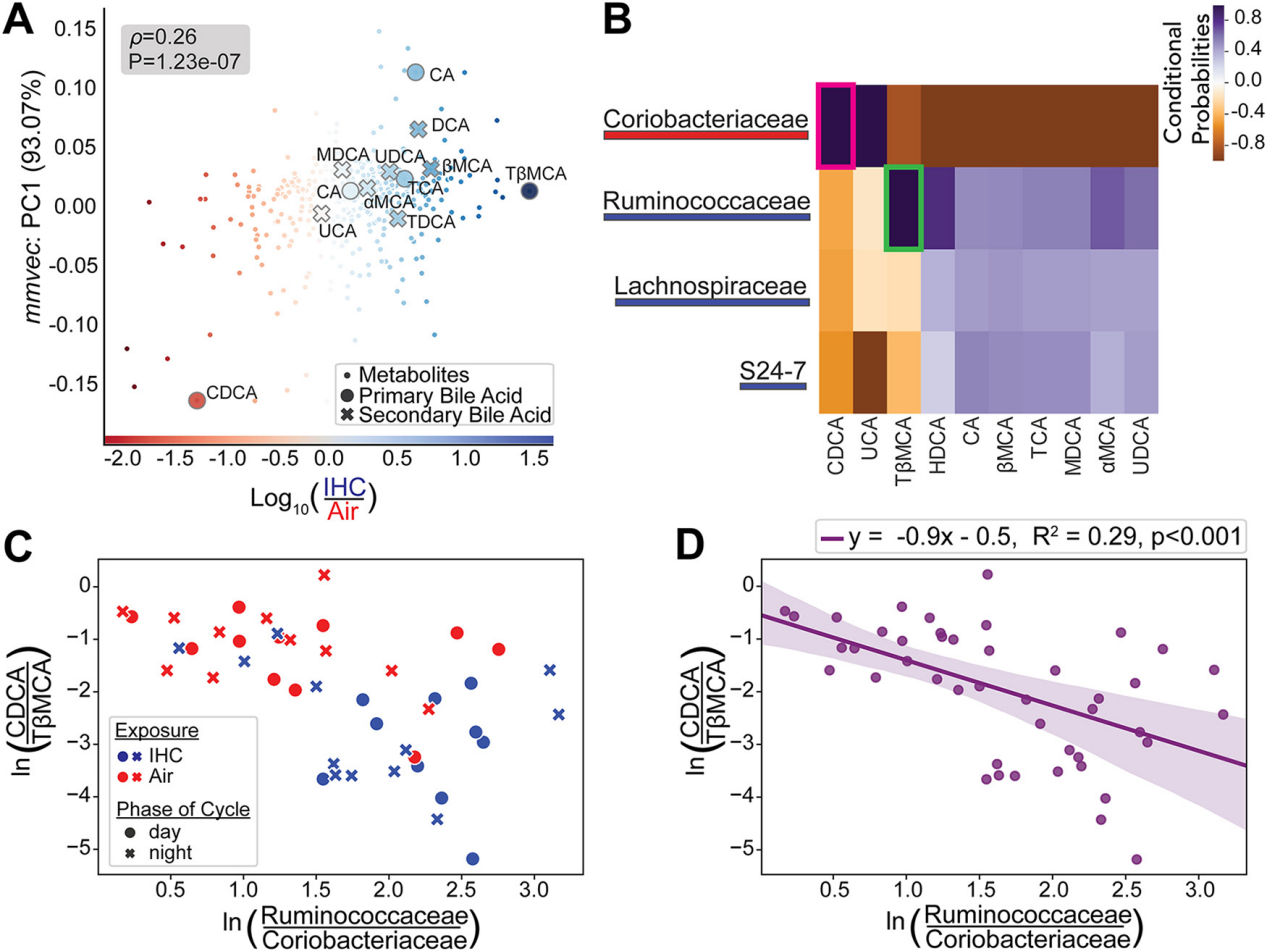
Microbes and metabolites with linked expression levels as determined by mmvec analysis. (A) mmvec (58) cooccurrence analysis (*y* axis) based on songbird (56) multinomial regression differential ranking analysis (*x* axis). Bile acids generally have level 1 identifications, except for cholic acid (CA), CDCA, and murideoxycholic acid (MDCA), which are level 3 annotations. (B) Log conditional probability heat map, organized using hierarchical clustering, with the top 4 differentially abundant microbial families and the top differentially abundant bile acids. Pink and green boxes highlight the top 2 points with the highest correlation values. (C) Log ratios of the top correlated microbes (*x* axis) and metabolites (*y* axis) identified in panel B. Microbial log ratios were determined as the number of all reads from sOTUs that belong to the family *Ruminococcaceae* divided by the number of all reads from sOTUs that belong to the family *Coriobacteriaceae*. Metabolite log ratios were determined as the raw values from CDCA divided by the raw values of TβMCA. (D) Linear regression plot using the same log ratios as the ones in panel C, with best-fit lines and shaded areas representing 95% confidence intervals. Log ratios are based on natural log. Control samples with exposure only to normal air conditions are in red (*n* = 4; 5 to 6 time points per mouse). Experimental samples exposed to IHC conditions are in blue (*n* = 4; 5 to 6 time points per mouse). Complete metadata can be found in Table S3 at https://doi.org/10.6084/m9.figshare.14614434.

Based on this multi-omic analysis, chenodeoxycholic acid (CDCA) and TβMCA are the two bile acids most differentially abundant between the IHC and control conditions (Fig. 3A). CDCA, which is associated with the air conditions, cooccurred with *Coriobacteriaceae* (Fig. S2G), and TβMCA, which is associated with the IHC conditions, cooccurred with *Ruminococcaceae* (Fig. 3A and B). Plotting the microbes and metabolites identified by mmvec validated its cooccurrence status (Fig. 3A and B). The log ratios of the top differentially cooccurring microbes and metabolites result in the separation of the two conditions. However, further studies are necessary to determine whether these relationships are causal. We found a significant correlation (Pearson’s correlation coefficient, *r*, = −0.539; *R*^2^ = 0.29; *P* < 0.001) between the log ratios of the mmvec-identified metabolites (CDCA under the control conditions and TβMCA under the IHC conditions) and the mmvec-identified microbes (*Coriobacteriaceae* under the control conditions and *Ruminococcaceae* under the IHC conditions) (Fig. 3C and D). Hence, after 1 week of IHC exposure, the cyclical dynamics of the luminal environment show significant shifts in the fecal microbiome and metabolome. The shift toward FXR antagonism via TβMCA and correlated with *Ruminococcaceae* is correlated with the pathophysiology of OSA-related CVD.

**FIG 4:**
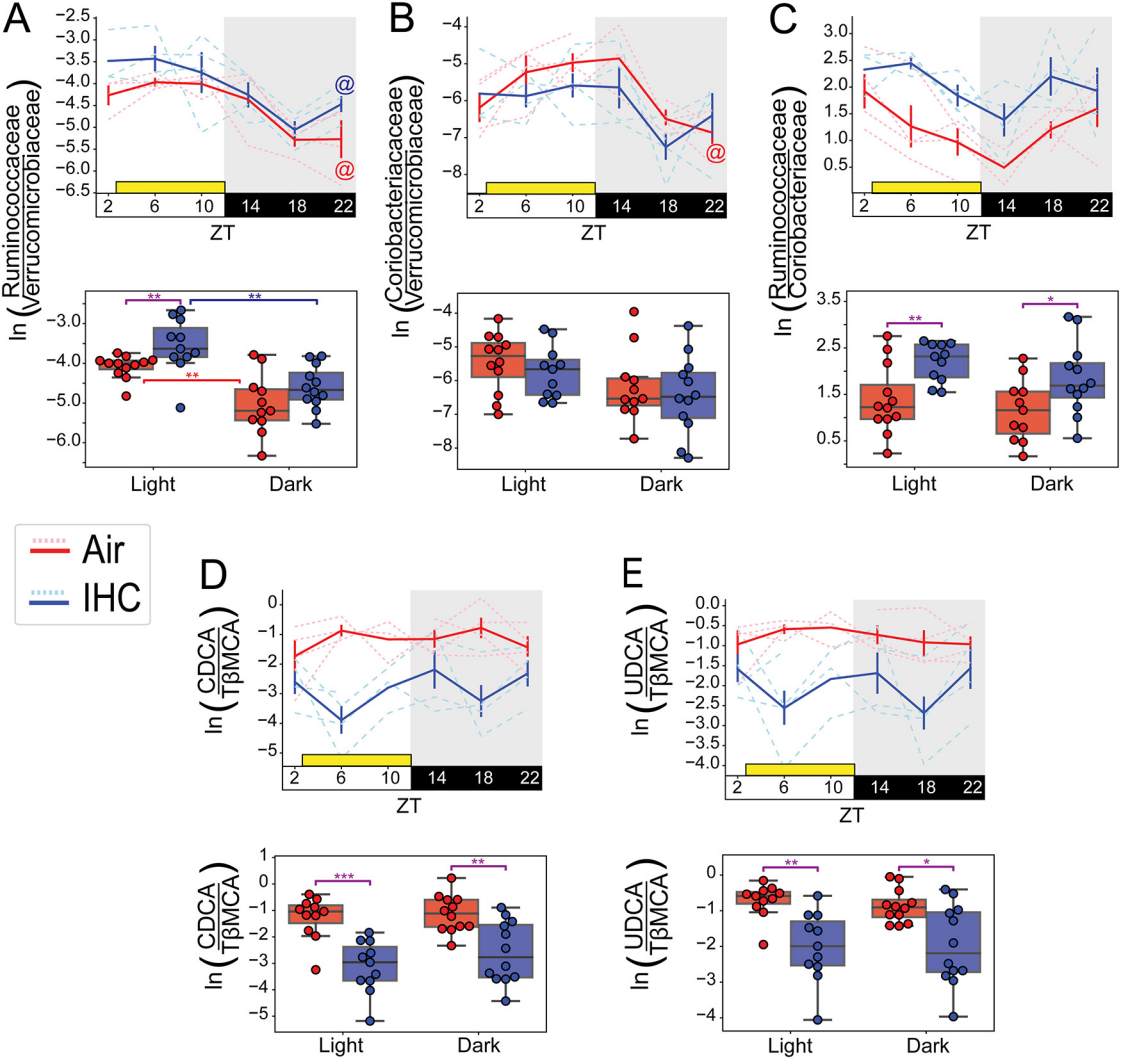
Cyclical dynamics of log ratios of key microbes and metabolites. Additional selected log ratios (natural log), their cyclical dynamics over time (double-line plots) (top), and their relative abundances grouped by cycle phase (box plots) (bottom) are shown. (A) Log ratios of all reads from sOTUs that belong to the family *Ruminococcaceae* divided by all reads from sOTUs that belong to the family *Verrucomicrobiaceae*. (B) Log ratios of all reads from sOTUs that belong to the family *Coriobacteriaceae* divided by all reads from sOTUs that belong to the family *Verrucomicrobiaceae*. (C) Log ratios of all reads from sOTUs that belong to the family *Ruminococcaceae* divided by all reads from sOTUs that belong to the family *Coriobacteriaceae*. (D) Log ratios of raw values of CDCA divided by raw values of TβMCA, the two most differentially abundant bile acids identified in Fig. S5B in the supplemental material. (E) Log ratios of the raw values of UDCA divided by the raw values of TβMCA. Solid lines represent the means, and error bars indicate standard errors of the means. Individual mice are indicated by dashed lines. Shading indicates when room lights are off (i.e., active/feeding time for the mice). Yellow squares indicate the 10 h of the day where mice under the IHC conditions would be exposed to experimental conditions (ZT2 [after collection] until ZT12). MetaCycle with the JTK method was used to determine cyclicity. *, *P* < 0.05; **, *P* < 0.01; ***, *P* < 0.001; ****, *P* < 0.0001 (by a Mann-Whitney-Wilcoxon test). @ indicates diurnal oscillations as determined by MetaCycle (JTK) with a *P* value of <0.05. Control samples with exposure to only normal air conditions are in red (*n* = 4; 5 to 6 time points per mouse). Experimental samples exposed to IHC conditions are in blue (*n* = 4; 5 to 6 time points per mouse). Error bars were not placed for time points where there were fewer than 3 log ratios available.

However, relative-abundance analyses did not show a significant difference between IHC and control conditions in these bacterial families (Fig. S2D and G). To determine whether total feature load differences could have biased these results, we repeated our assessment of these two bacterial families using log ratios. For the denominator of these log ratios, we used the family *Verrucomicrobiaceae* due to its ubiquity and high abundance. As predicted by the differential ranking analysis (Fig. 3B), mice under the IHC conditions had higher log ratios of *Ruminococcaceae* to *Verrucomicrobiaceae*, especially during the light period (Fig. 4A). Conversely, mice under the air conditions had higher log ratios of *Coriobacteriaceae* to *Verrucomicrobiaceae* during the light/inactive period (Fig. 4B). Log ratios of *Ruminococcaceae* to *Coriobacteriaceae* show persistently higher levels of *Ruminococcaceae* in mice under IHC conditions, whereas mice under control conditions have higher relative levels of *Coriobacteriaceae* (Fig. 4C).

Log ratio analysis of the two most different bile acids (i.e., CDCA and TβMCA) also showed clear differences between IHC and control mice. As predicted by the differential ranking analysis (Fig. 3A and B), mice under the IHC conditions have far more fecal TβMCA relative to CDCA than mice under the control conditions (Fig. 4D). Interestingly, CDCA is an FXR agonist, while TβMCA is an FXR antagonist. FXR is a bile sensor that regulates bile acid uptake, metabolism, and excretion that can affect atherosclerosis (47). Although the mmvec analysis did not identify UDCA, a secondary bile acid with anti-inflammatory properties (49, 51, 52), as being associated with any specific bacterial family or any condition, we repeated the log ratio analysis with this secondary bile acid. In this case, mice under the IHC conditions have far more of the proatherosclerotic bile acid TβMCA relative to UDCA than mice under the control conditions (Fig. 4E).

## DISCUSSION

In this study, we demonstrate that the compositional and diurnal dynamics of the microbiome and metabolome of atherogenic *Apoe^−/−^* mice are significantly impacted by IHC conditions. Despite the low number of mice used in this study (*n* = 4), we have found significant differences in both microbial families and metabolites at different time points. IHC exposure leads to significant changes in diurnal oscillations of secondary metabolites that are key contributors to the pathogenesis of atherosclerosis. It is currently unclear if a gain or loss of oscillations is more important for disease outcomes based on the literature. Taken together, IHC result in circadian dyssynchrony of the gut microbiome and metabolome, which promotes a proinflammatory luminal environment through which atherosclerosis is exacerbated. Moreover, it suggests that bile acid signaling and disturbed peripheral circadian rhythms likely contribute to the IHC-induced increase in cardiovascular risk.

Several studies, in both mice and humans, demonstrate the metabolic implications of disruptions to the circadian homeostasis of the microbiome (7, 8). In humans, jet lag-induced disruption of microbiome diurnal dynamics leads to increased adiposity in gnotobiotic mice (10). More recently, a large-scale epidemiological study demonstrated that circadian dyssynchrony of microbial oscillations was associated with type 2 diabetes. Moreover, these arrhythmic microbial risk signatures were highly predictive of metabolic disease (59). Disruption of the circadian dynamics of the microbiome is a hallmark of animal models of obesity and dysmetabolism (9–11). In this study, we show that diurnal disruption of the gut microbiome is also a hallmark of preclinical models of OSA. Since the gut microbiome is necessary to entrain peripheral circadian rhythms, OSA could contribute to dysmetabolism by inducing circadian dyssynchrony. This finding could explain why OSA can increase CVD risk across so many different physiological systems (e.g., hypertension and insulin resistance).

Our previous work in preclinical models of OSA demonstrates that IHC induce reproducible microbiome and metabolome changes across two different mouse models of atherosclerosis, *Apoe^−/−^* and *Ldlr^−/−^* mice (29). These luminal changes were predictive of IHC exposure and could be used potentially to highlight atherosclerotic risk (26). Despite measuring the microbiome and metabolome composition only 1 week after the initiation of daily IHC intervention, we found similar changes in this study. Moreover, since the study was focused solely on OSA and the impact of IHC, we used the most well-defined model (*Apoe^−/−^* mice on an atherogenic diet) and determined the impact of a single factor (environmental changes in gases) that exacerbates atherosclerosis and whether it impacts the dynamics of the gut microbiome. Since genetics and diet were adequately controlled for under our experimental and control conditions, there is no need to disentangle their effects from the observed effects of IHC. Although the circadian impacts of genetics and diet have not yet been investigated in the context of atherosclerosis, several studies have shown that they impact diurnal dynamics of the gut microbiome (25–28). Experiments were performed using best-practice guidelines to minimize maternal, founder, and cage effects (60–62). In addition, all mice were from the same source vivarium, room, and maternal line (to minimize maternal effects); were acclimated in shared holding cages in the vivarium (to control for founder effects); and were then pseudorandomized into individually housed cages (to control for cage effects).

IHC imposed microbiome compositional changes that are often observed in dysmetabolic states, including changes in the diversity and abundances of specific bacterial families. IHC-induced reduction of microbial diversity and richness occurred within 1 week of exposure. This observation suggests that IHC make the lumen uninhabitable for many commensal microbes, likely by changing environmental microniches. Moreover, these changes are not restricted to only the time of exposure to IHC (i.e., light period); there are global shifts in the gut microbiome even during the times of the day when the animal is not being exposed to IHC. Interestingly, the dark-period microbiome of the IHC is more similar to the light-period microbiome composition of the control mice. Importantly, changes to the microbiome oscillations occur almost immediately, within 1 week of IHC exposure, confirming that this change results from IHC, rather than the atherosclerotic phenotype, which can take 10 weeks to develop (25, 26, 29). Importantly, these changes can create a lasting and profound impact on the metabolic health of the host.

Previous studies investigating diurnal cycling of the gut microbiome have used samples collected under 24-h (9, 11, 13) and 48-h (10, 12, 63) conditions. Although in general, it is preferable to use 48-h data for circadian studies since it reduces type I errors, recent advances in bioinformatic tools, such as MetaCycle (35), allow rigorous analysis of 24-h circadian data. This allows investigators to determine circadian/diurnal cycling from more limited data while still reducing type I errors. Although these tools were created for transcriptional data, as opposed to microbiome data, since our work ultimately replicates and expands upon the results of previous microbiome IHC studies (64) and is consistent with diurnal microbiome studies, these potential issues do not significantly impact interpretation. Nevertheless, a more thorough study comparing 24-h and 48-h diurnal microbiome data is warranted to determine if MetaCycle is as robust for this type of data as it is for transcriptional data.

IHC significantly affected the diurnal dynamics of the gut microbiome. *Verrucomicrobia*, *Firmicutes*, and *Bacteroidetes*, phyla that composed more than 90% of the gut microbiome, were all affected by the IHC conditions. The relative abundances of families implicated in atherosclerosis, *Ruminococcaceae* and *Lachnospiraceae* (22, 65), had circadian oscillations in control mice but lost these oscillations in mice exposed to IHC conditions. In particular, the family *Lachnospiraceae*, a bacterial family that has been associated with increases in proatherosclerotic TMA *N*-oxide (TMAO) and an increase in a thrombotic phenotype, was significantly elevated in IHC mice during the dark period (66). IHC also led to the relative reduction of Akkermansia muciniphila, the only microbe in the phylum *Verrucomicrobia* found in the gut luminal environment of mammals. A. muciniphila is crucial for gut barrier integrity, which helps prevent a proinflammatory state by impeding the translocation of luminal compounds into the portal system (67). Replenishing these species in the microbiome of Western-diet-fed *Apoe^−/−^* mice resulted in a decrease in atherosclerotic lesions (68). Furthermore, IHC induced a gain of oscillation in S24-7, a family in the *Bacteroidetes* phylum. This family had significantly increased relative abundances during the light period. Some members of S24-7 contain an SpeB homolog, a cysteine protease (34), which helps these bacteria avoid detection by the immune system (34, 69) and can potentially degrade the protective biofilm present on the surface of the mucosal layer (70). Together, the increase in S24-7 and decrease in *A. muciniphila* suggest a disruption of the host mucosal layer and a breakdown of the gut barrier function. Whether these microbiome changes are the sole cause of the IHC-induced increase in CVD risks or exacerbators of the dysmetabolic phenotype warrants further investigation.

Along with changes to the gut microbiome, the IHC-conditioned metabolome was also altered with increased levels of proinflammatory metabolites, particularly bile acids. A comparative analysis of dark-period versus light-period fecal samples revealed higher levels of proinflammatory and proatherosclerotic bile acids, such as DCA and TβMCA, in IHC mice regardless of the time of collection. Moreover, atherosclerosis studies that collect fecal pellets during the light period for metabolomics analysis may underestimate their potential role in CVD risk associated with IHC. DCA gains circadian oscillation in IHC mice, with the most significant changes from controls occurring during the dark period. Elevated fecal DCA boosts overall systemic inflammation (71–73), which in IHC may be especially problematic in the context of a potentially disrupted intestinal barrier. TβMCA, a naturally occurring FXR antagonist (46), was also significantly elevated in IHC, especially during IHC exposure. There is conflicting evidence on the role of FXR in atherosclerosis development and progression, and thus, it is possible that these proinflammatory changes are protective as opposed to pathogenic. However, deletion of FXR in *Apoe^−/−^* mice results in lesion exacerbation (48), suggesting that FXR antagonism with excessive TβMCA contributes to worse CVD under the IHC conditions in our preclinical model of OSA. In addition, anti-inflammatory and antiatherosclerotic bile acids (e.g., UDCA) were decreased under IHC conditions.

Overall differences in bile acid levels between the IHC and control groups were more pronounced during the dark period. Moreover, our trans-omic analysis (mmvec plus ranked multinomial regression), which divulges relationships between luminal microbial composition and luminal metabolites that are related to our experimental conditions, resulted in conditional probabilities with two key findings. First, the abundance of *Ruminococcaceae* cooccurs with TβMCA. Many members of the *Ruminococcaceae* have 7α-dehydroxylation and 7β‐dehydrogenation genes that help them perform bile acid biotransformations (74, 75). In fact, the *Ruminococcaceae* family is positively correlated with fecal DCA levels (76), which it likely helps create with 7α-dehydroxylation. Increased levels of both *Ruminococcaceae* and TβMCA were associated with IHC exposure, further highlighting the presence of a proinflammatory, proatherosclerotic environment under these conditions. Second, our trans-omic analysis found that the abundance of *Coriobacteriaceae* cooccurred with CDCA. Increased levels of both *Coriobacteriaceae* and CDCA were associated with mice under the control conditions. FXR agonism with CDCA protects against dyslipidemia and atherosclerosis in *Apoe^−/−^* mice (77). Further investigation is required to understand how CDCA and *Coriobacteriaceae* may be linked. *Coriobacteriaceae* have been thought to have beneficial metabolic effects (78), including resistance to obesity and liver pathologies (79).

Bile acids are not the only atherosclerosis-related metabolites that were affected by IHC. There were also significant increases in proatherosclerotic glycerolipids, including phosphocholines and phosphoethanolamines, under the IHC conditions. Phosphocholines are known to be components of LDL (“bad”) cholesterol and interact with C-reactive protein in a proinflammatory and proatherosclerotic manner (80–82). Increased excretion of bilirubin breakdown products, stercobilin and urobilin, under IHC conditions may be an early biomarker of liver dysmetabolism (43). Interestingly, IHC mice had significantly decreased levels of acylcarnitines during both phases of the day compared to control mice. This is the only proatherosclerotic metabolite that we measured that was decreased under the IHC conditions. Since changes in the microbiome suggest increased gut permeability, decreased levels of acylcarnitines in stool may indicate increased absorption into the serum, where they promote inflammation and are associated with an increased risk of myocardial infarction (83), or this may indicate that IHC-induced atherosclerosis is not driven by acylcarnitines. Future studies will need to determine the relationship between fecal and serum acylcarnitines to help determine if this is the case.

One of the limitations of this study is that, due to methodological constraints, we were not able to assess the levels and diurnal oscillations of known proatherosclerotic and anti-inflammatory small molecules, such as TMA (which is then converted to TMAO in the liver) and SCFAs, respectively. Cyclical fluctuations in SCFAs have been documented in control mice as well as those with dysmetabolic phenotypes, and SCFAs can affect hepatic peripheral circadian rhythms (8). Given the extent of the IHC-induced diurnal changes in the luminal environment, there is little doubt that these metabolites likely fluctuate with OSA as well. Taken together, changes in the gut microbiome oscillations and the different types of metabolites have complex pathological implications that require further investigation.

Since IHC had a remarkable influence on the oscillations of the microbiome and metabolome in the luminal gut, and since IHC are composed of synchronous changes in O_2_ and CO_2_ in opposite directions, our results in this work indicate that each specific gas change may have an influence on the cyclicity of the gut microbiome. On the other hand, perturbed luminal dynamics suggest altered nutrient availability. IHC may disrupt normal feeding patterns, likely due to increased stress or lack of sleep in these mice. Normal feeding patterns are essential for the maintenance of the peripheral circadian clock. Time-restricted feeding (TRF), where a normal feeding pattern is maintained by consolidating access to food only to the active time period, enforces central and peripheral circadian clock synchrony and prevents dysmetabolic phenotypes in a number of nutritional and nonnutritional challenges to metabolic homeostasis (7, 84–86). Whether TRF can protect against IHC-induced CVD risks is yet to be determined. Moreover, understanding how bile acid modifications performed by the gut microbiome modulate host metabolic mechanisms will provide valuable insight into the pathophysiology of IHC-induced atherosclerosis. Although there is sufficient evidence that bile acid signaling plays an important role in atherosclerosis, a better understanding of bacterial bile acid biotransformations and their contribution to IHC-induced pathogenesis will yield novel therapeutic targets. Since the changes in bile acids found in this study are centered around FXR expression, future experiments should further investigate the role of bile acid receptors (i.e., FXR and TGR5) in mediating the effects of IHC on host atherosclerosis. Importantly, our study clearly demonstrates the importance of considering the time of sample collection in the experimental design, as many of the differences observed in our study would have gone unnoticed if samples were collected at a single time point or only during the light period.

## MATERIALS AND METHODS

### Animal model and description of IHC induction.

Individually housed, 10-week-old *Apoe^−/−^* mice (littermates from Jackson Laboratories) were fed an irradiated regular chow diet (catalog number LB-485, diet number 7912; Envigo Teklad) prior to the start of the experiment. Upon starting the experiment, the mice were fed an atherogenic diet consisting of 1.25% cholesterol and 21% milk fat (4.5 kcal/g) (catalog number TD.96121; Envigo-Teklad, Madison, WI) *ad libitum*. There were 8 mice in total, with 4 mice pseudorandomly assigned to each of 2 groups (based on weight and acclimation cage). The vivarium was maintained in a 12-h–12-h light-dark-cycle room at 68°F to 72°F and below 40% humidity during the study. IHC exposure occurred as previously described (25). In brief, mice were exposed to 10 h of IHC conditions during the light period (ZT2 to -12). IHC exposure consisted of 4 min of a synchronized reduction of O_2_ from 21% to 8% with a synchronized elevation of CO_2_ from 0.5% to 8%, followed by alternating periods of 4 min of normoxia and normocapnia with 1- to 2-min ramp intervals. Control mice were kept in room air (21% O_2_ and 0.5% CO_2_) for the duration of the experiment. The introduction of both the atherogenic diet and IHC conditions commenced on day 0. The 1-week time point was chosen so that the mice would have several days to adjust to the IHC chamber. We wanted to focus on early environment-induced changes, which drive the phenotype, rather than later in phenotype development, where the effects of the dysmetabolic state could affect the gut microbiome composition. All animal experiments were conducted in accordance with the guidelines of the IACUC of the University of California, San Diego.

After 1 week of maintaining the mice in their relative environmental conditions, fecal samples were collected every 4 h (ZT2, -6, -10, -14, -18, and -22) over a 24-h period. After collection, fecal samples were immediately stored at −80°C until the end of the study. The microbiome was characterized by 16S rRNA amplicon sequencing, and the metabolome was characterized by untargeted liquid chromatography-tandem mass spectrometry (LC-MS/MS) in a manner consistent with those of previous studies (25, 26).

### Characterization of the microbiome.

DNA extraction and 16S rRNA amplicon sequencing were done using the standard protocol for the Earth Microbiome Project (https://earthmicrobiome.org/protocols-and-standards/) (87). In brief, DNA was extracted using the Qiagen PowerSoil DNA extraction kit (Qiagen, Carlsbad, CA). The resulting DNA library was prepared for 16S rRNA amplicon sequencing as described previously (87). These pooled samples were purified using a Qiagen UltraClean PCR cleanup kit (Qiagen, Carlsbad, CA) and then sequenced on the Illumina HiSeq 2500 sequencing platform. The V4 region of the 16S rRNA gene was sequenced using the primer pair 515F-806R with Golay error-correcting barcodes on the reverse primer (88).

Raw sequence data were uploaded to Qiita (89) (https://qiita.ucsd.edu/) and processed using the Deblur (90) workflow with default parameters. There were 2,490,504 reads for 94 samples with an average of 28,519 reads per sample. This process generated a BIOM (91) format table that was rarefied to a depth of 12,000 sequences/sample to control for sequencing effort. This process removed two samples from the analysis, one from air (ZT2) and one from IHC (ZT14), that had read counts similar to those of the blanks. A rooted phylogenetic tree was inferred using the SATé-enabled phylogenetic placement plug-in (92) using QIIME 2 (93) version 2019.10, which was used to insert 16S Deblur sub-operational taxonomic units (sOTUs) into Greengenes (94) 13_8 at 99%. Because it takes into account both phylogeny and abundance, weighted UniFrac (95) distances were used for microbiome principal-coordinate analysis (PCoA) plots. Overall group differences were tested using PERMANOVA (33). All sOTUs were collapsed to the phylum and family levels for analysis and comparison because critical sOTUs that distinguish IHC and air from each other did not identify past the family level. MetaCycle using the JTK method and correction for multiple hypotheses with Fisher’s method was applied to determine circadian/diurnal oscillatory patterns (35). Rhythmicity was tested using LimoRhyde (36). A Mann-Whitney-Wilcoxon test was used to compare groups presented in the box plots. Data were visualized using custom Python scripts (the Python code is available at https://github.com/knightlab-analyses/circadian-ihc/).

### Characterization of the metabolome.

Untargeted LC-MS/MS was performed on the stool samples for metabolomics as previously described (25). In brief, samples were prepared by adding 500 μl of 50:50 methanol-H_2_O to all fecal samples (approximately 30 to 50 mg), followed by homogenization and centrifugation. Next, 450 μl of the resulting supernatant was transferred to a 96-well deep-well plate and dried using centrifugal evaporation (CentriVap centrifugal vacuum concentrator; Labconco, Kansas City, MO). Next, samples were resuspended, and samples were analyzed on a Vanquish ultrahigh-performance liquid chromatography (UPLC) system coupled to a Q Exactive orbital ion trap (Thermo Fisher Scientific, Bremen, Germany). For chromatographic separation, a C_18_ core shell column (Kinetex column, 50 by 2 mm, 1.7-μm particle size, 100-Å pore size; Phenomenex, Torrance, CA) with a flow rate of 0.5 ml/min (solvent A, H_2_O–0.1% formic acid [FA]; solvent B, acetonitrile–0.1% FA) was used. Flowthrough parameters were set and run, and data were collected as previously described (25).

The raw data resulting from the method described above were converted to *m/z* extensible markup language (mzXML) in centroid mode using MSConvert (part of ProteoWizard) (96). The mzXML files were cropped using an *m/z* range of 75.00 to 1,000.00 Da for further sample processing. Using a signal threshold of 2.0e5 and a 0.3-s minimum peak width to remove low-quality spectra, MZmine2 (97) (http://mzmine.sourceforge.net/) was used for feature extraction. The local minimum search algorithm was used with a minimum relative peak height of 1% for chromatographic deconvolution. The minimum retention time range was set to 0.6 s. The maximum peak width was set to 1 min. After that, alignment of the peak lists of all samples was performed with a retention time deviation of 10 s, and a mass tolerance of 10 ppm was set for features. Next, MZmine2 (97) (version 2.37) was used to create a feature matrix file that could then be linked to the metadata. The signal intensities of the features were normalized for subsequent analysis. Identification of molecular features was performed using MS1-based feature detection and MS2-based molecular networking using the GNPS (98) workflow (https://gnps.ucsd.edu/ProteoSAFe/static/gnps-splash.jsp). The actual GNPS jobs can be found at https://gnps.ucsd.edu/ProteoSAFe/status.jsp?task=9d8ee19ec2654d46a065080a0ff2290a. Using a decoy database approach in GNPS, we determined that the false discovery rate (FDR) was less than 1% above a 0.6 cosine similarity score (99). Thus, we used a cosine score of 0.65 to determine annotations. The MS/MS spectral annotations were determined by using MS/MS-based spectral library matches for GNPS level 2 or 3 annotations for all molecules (100). Bile acid standards were purchased from Cayman Chemical (Ann Arbor, MI) and analyzed using the same LC-MS/MS method as the one described above to attain level 1 identification as defined by the 2007 Metabolomics Standards Initiative (101). The annotated frequency table was analyzed using QIIME 2 (93) version 2019.10. Canberra distances (102), which are more sensitive to rare features than Bray-Curtis distances, were used for metabolomic PCoA plots, and significance was tested using PERMANOVA (33). MetaCycle (35) utilizing the JTK method was used to determine circadian/diurnal rhythmicity. A Mann-Whitney-Wilcoxon test was used to compare groups presented in the box plots. Data were visualized using custom Python scripts (the Python code is available at https://github.com/knightlab-analyses/circadian-ihc/).

### Differential abundance and multi-omic analyses.

Differential abundance analysis was performed with songbird, which accounts for the compositional nature of microbial data and uses a multinomial regression model to estimate differential ranks (56). Optimized model parameters for the covariate of exposure treatment and the interaction of time in hours were determined for the microbiome and metabolomics data (differential prior of 1.5 and learning rate of 0.001) and compared to a baseline model of 1 on the same parameters. The model fits were compared by the *Q*-squared (1 − model coefficient of variation [CV]/baseline CV) value, where a *Q*-squared value of >0 ensures a good model fit. *Q*-squared values of 0.14 and 0.22 were obtained for the microbiome and metabolite data, respectively. The resulting differentials were explored and verified by log ratios through Qurro (103). Multi-omic analyses of microbiome and metabolomics data were performed through mmvec (microbe-metabolite vectors), a neural network method for producing log-conditional probabilities of cooccurrence between microbial and metabolite features visualized as heat maps and paired latent representations in few dimensions, which can be visualized in scatterplot or biplot ordinations (58). A high conditional probability of close spatial similarity in the ordination indicates high cooccurrence between a microbe and metabolite pair, while a negative conditional probability or a high spatial distance in the ordination indicates low cooccurrence. The mmvec model parameters were optimized (batch size of 5 and learning rate of 1e−3) to minimize the low cross-validation error and model likelihood. Differential cooccurrence patterns (mmvec microbe-metabolite interactions) in relation to the exposure treatment were evaluated by correlating the mmvec PC1 loading with respect to the songbird log fold change differential with respect to the exposure treatment, which was significantly correlated (metabolites) (Spearman rho = 0.26; *P* = 1.23e−07) (see Results).

### Data availability.

The EBI accession number for the microbiome is ERP110592; the MassIVE identifier for the metabolome is MSV000084847. The Python code is available at https://github.com/knightlab-analyses/circadian-ihc/.
