# Supplementary figures and images for: Intermittent Hypoxia and Hypercapnia Alter Diurnal Rhythms of Luminal Gut Microbiome and Metabolome
Source: mSystems. 2021 Jun 29;6(3):e00116-21. doi: 10.1128/mSystems.00116-21 (PMC8269208; doi:10.1128/mSystems.00116-21)

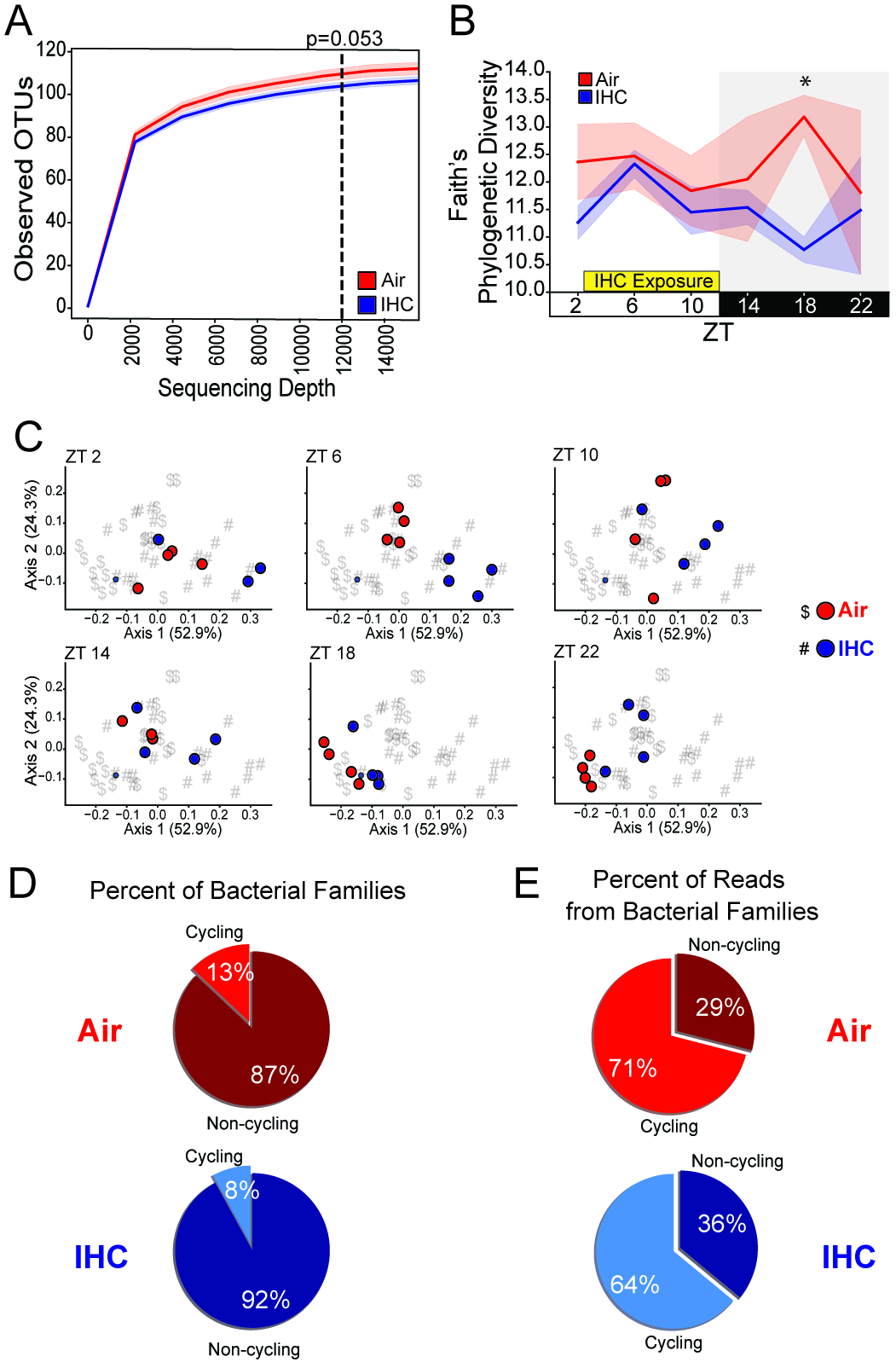

Supplement: FIG S1 [file msystems.00116-21-sf001.tif]

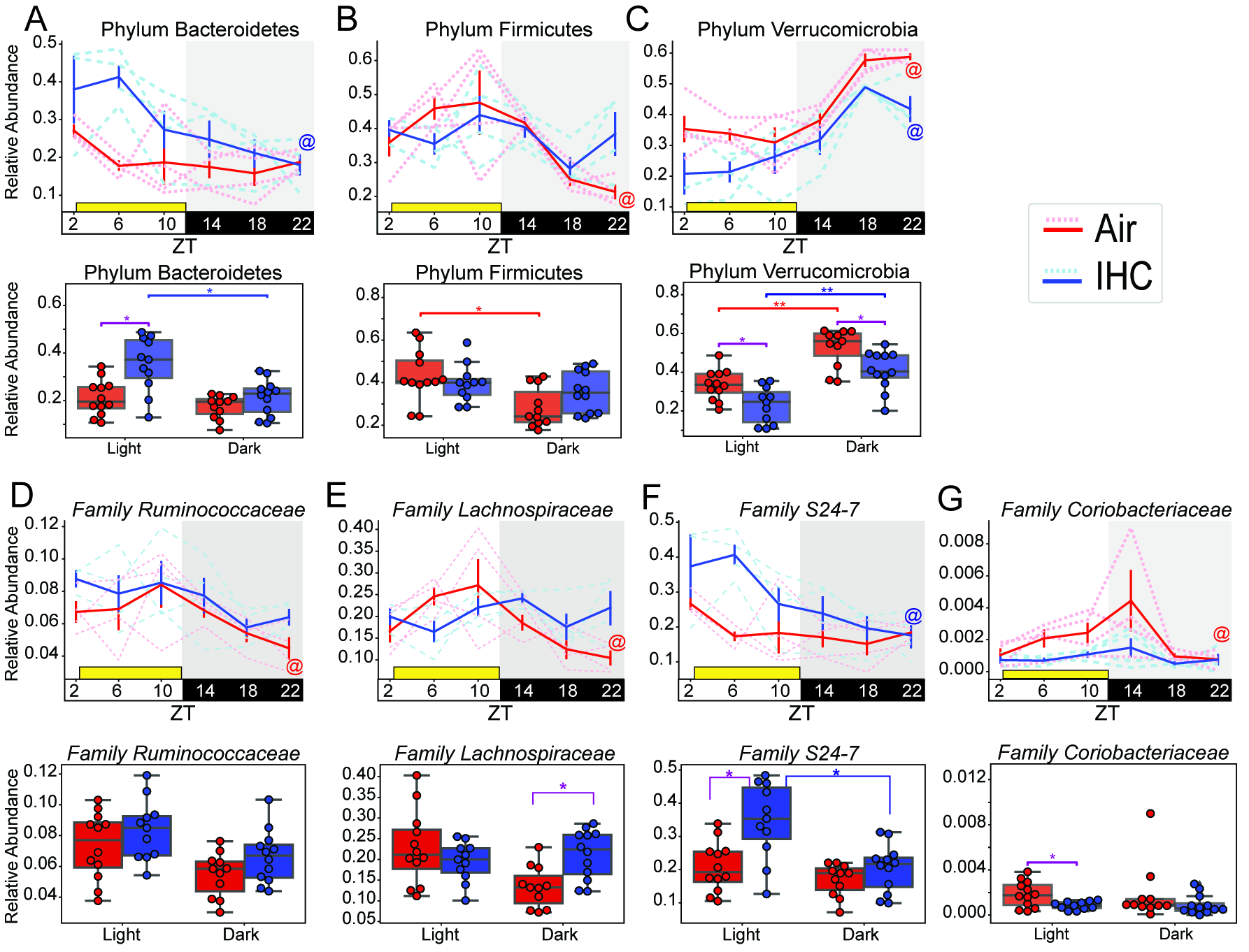

Supplement: FIG S2 [file msystems.00116-21-sf002.tif]

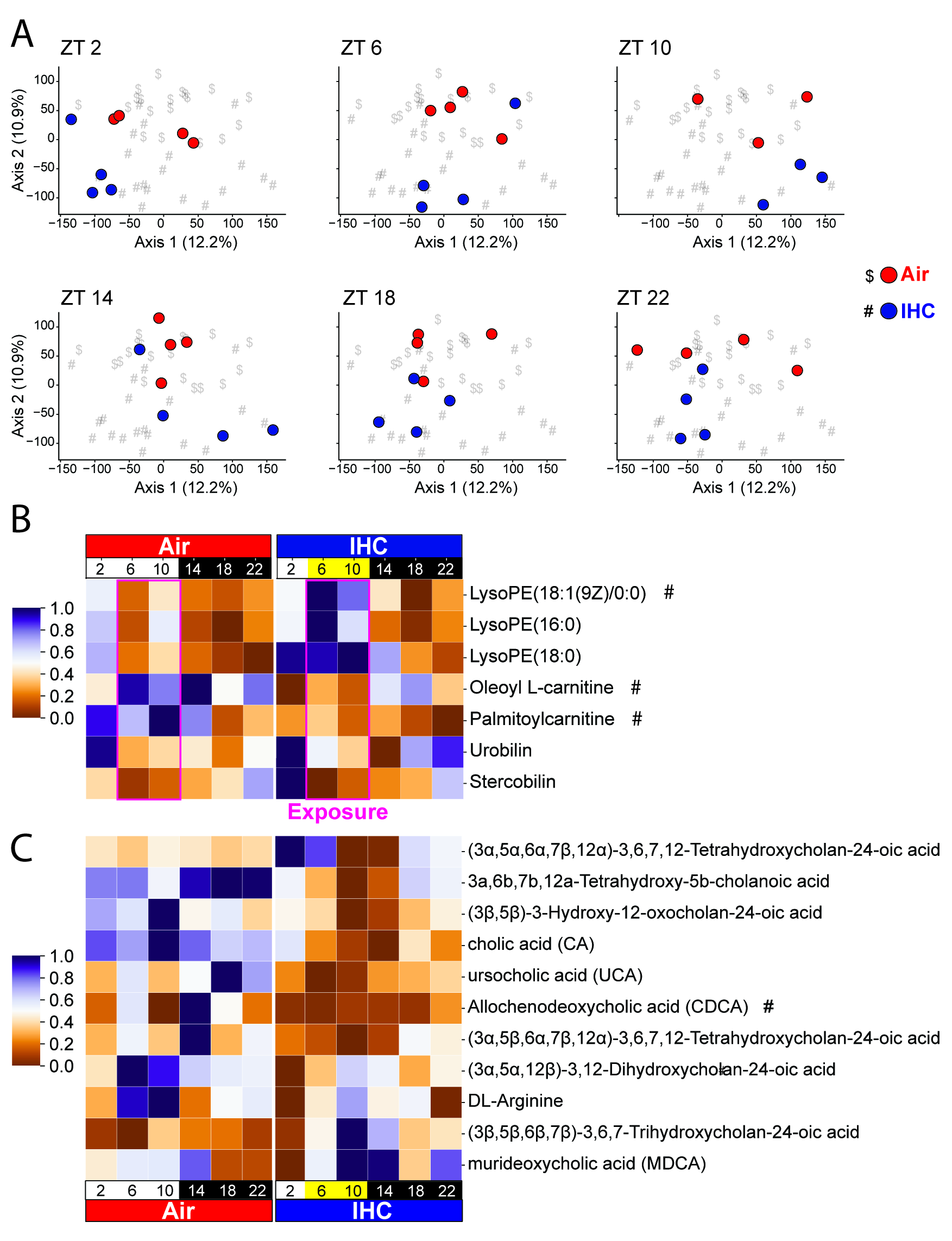

Supplement: FIG S3 [file msystems.00116-21-sf003.tif]

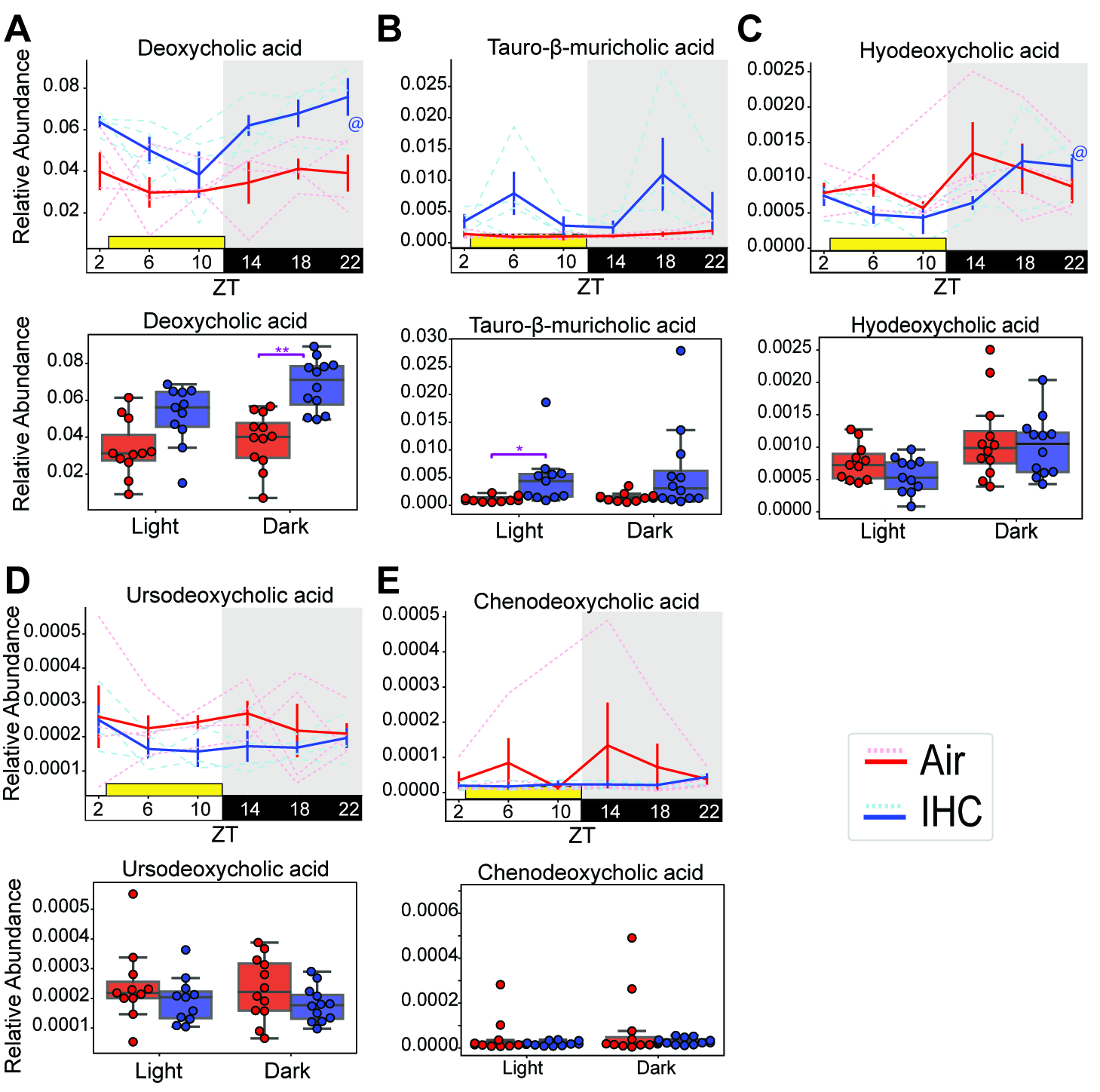

Supplement: FIG S4 [file msystems.00116-21-sf004.tif]

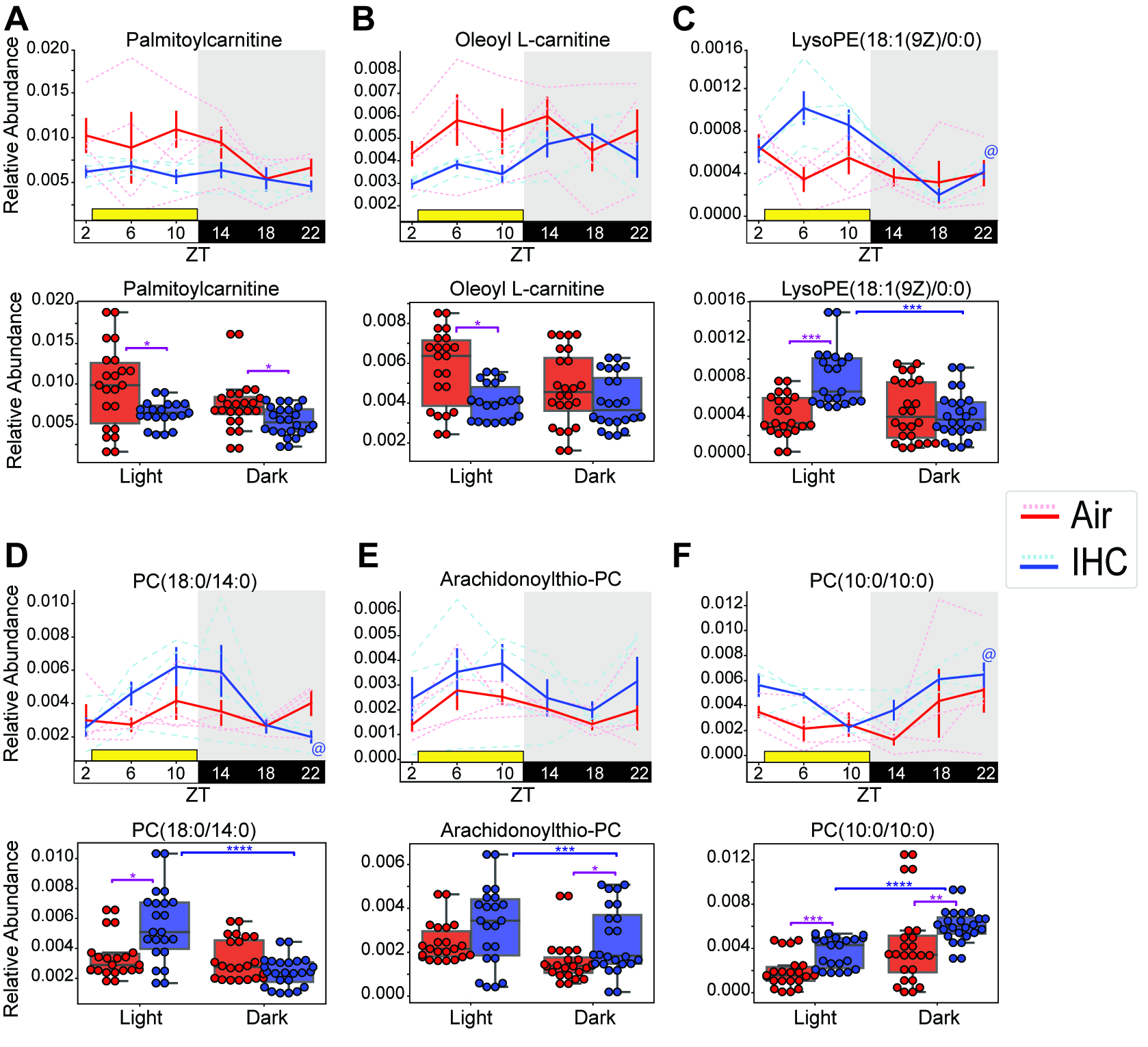

Supplement: FIG S5 [file msystems.00116-21-sf005.tif]
